# Links between Dietary Protein Sources, the Gut Microbiota, and Obesity

**DOI:** 10.3389/fphys.2017.01047

**Published:** 2017-12-19

**Authors:** Lise Madsen, Lene S. Myrmel, Even Fjære, Bjørn Liaset, Karsten Kristiansen

**Affiliations:** ^1^National Institute of Nutrition and Seafood Research, Bergen, Norway; ^2^Laboratory of Genomics and Molecular Biomedicine, Department of Biology, University of Copenhagen, Copenhagen, Denmark; ^3^BGI-Shenzhen, Shenzhen, China

**Keywords:** gut microbiota, obesity, dietary proteins, diet, protein source, mouse models, metabolism, dietary fats

## Abstract

The association between the gut microbiota and obesity is well documented in both humans and in animal models. It is also demonstrated that dietary factors can change the gut microbiota composition and obesity development. However, knowledge of how diet, metabolism and gut microbiota mutually interact and modulate energy metabolism and obesity development is still limited. Epidemiological studies indicate an association between intake of certain dietary protein sources and obesity. Animal studies confirm that different protein sources vary in their ability to either prevent or induce obesity. Different sources of protein such as beans, vegetables, dairy, seafood, and meat differ in amino acid composition. Further, the type and level of other factors, such as fatty acids and persistent organic pollutants (POPs) vary between dietary protein sources. All these factors can modulate the composition of the gut microbiota and may thereby influence their obesogenic properties. This review summarizes evidence of how different protein sources affect energy efficiency, obesity development, and the gut microbiota, linking protein-dependent changes in the gut microbiota with obesity.

## Introduction

The importance of the gut microbiota in obesity is well documented (Villanueva-Millan et al., 2015; Sonnenburg and Backhed, 2016; reviewed in Baothman et al., 2016). Still, lifestyle and diet are closely linked to obesity, and increased consumption of energy-dense food with a concomitant increase in total energy intake is probably a major driver of the obesity epidemic (Swinburn et al., 2011; Romieu et al., 2017). In humans dietary patterns play important roles in shaping the gut microbiota (De Filippo et al., 2010; Muegge et al., 2011; Yatsunenko et al., 2012; David et al., 2014; Schnorr et al., 2014; Carmody et al., 2015; Graf et al., 2015) as well as in the development of obesity (Bellissimo and Akhavan, 2015).

High protein diets are reported to promote weight loss and weight maintenance in humans (Westerterp-Plantenga et al., 2009, 2012; Pesta and Samuel, 2014; Pasiakos, 2015), but a systematic review revealed that the long-term effects of high-protein diets are neither consistent nor conclusive (Lepe et al., 2011). The results from rodent trials are more consistent, and a number of studies has demonstrated that a high protein: carbohydrate ratio prevents high fat diet induced obesity (Lacroix et al., 2004; Marsset-Baglieri et al., 2004; Morens et al., 2005; Pichon et al., 2006; Madsen et al., 2008; Ma et al., 2011; Freudenberg et al., 2012, 2013; Hao et al., 2012; McAllan et al., 2014).

Importantly, the notion that the obesogenic potential of high fat diets in rodents is attenuated by increasing the protein:carbohydrate ratio is largely based on studies using casein or whey as the protein source. However, there is little knowledge as to how different protein sources may modulate the response to high protein intake. Recently, we demonstrated that feeding obesity-prone C57BL/6J mice a high fat, high protein diet using casein, soy, or filets of cod, beef, chicken (skinless) or pork as protein sources led to striking differences in obesity development at thermoneutral conditions. Casein was the most efficient protein source preventing weight gain and accretion of adipose mass, whereas mice fed high protein diets based on “white meat” (lean pork or chicken filets) had the highest increase in feed efficiency and adipose tissue mass (Liisberg et al., 2016b). Epidemiological studies also indicate that intake of dairy and vegetarian protein sources is associated with protection against obesity, whereas a high intake of meat, in particular red meat, predicts higher weight gain (Fogelholm et al., 2012; Smith et al., 2015; Mozaffarian, 2016). To what extent intake of red meat protein affects the composition and function of the gut microbiota in humans remains to be established.

It has been suggested that dietary patterns are associated with distinct bacterial communities in the human gut (Wu et al., 2011). It is further well known that dietary characteristics, such as the content of fat, whole grain, fruits and nuts, as well as high fiber diets in general, influence the gut microbiota (De Filippo et al., 2010; Muegge et al., 2011; David et al., 2014; Schnorr et al., 2014; Carmody et al., 2015; Graf et al., 2015; Eid et al., 2017). However, it is still not established which food constituents specifically promote growth and function of beneficial bacteria in the intestine. Recently, differences in profiles of gut bacteria between rats fed proteins from red meat (beef and pork), chicken, and fish (here defined as white meat) and other sources (casein and soy) were reported (Zhu et al., 2015, 2016). Still, very little is known about the effect of protein source and quality on the regulation of energy balance.

An undoubtedly important factor determining protein quality is the amino acid composition (Millward et al., 2008). Different protein sources provide different amino acids (AAs) that differ in properties and effects on body functions. In addition to the amino acid composition, different protein sources vary in the content of micro- and macronutrients as well as undesirable compounds. For instance, legumes are rich in fibers, red meat is generally rich in saturated fat and iron, dairy products are generally high in calcium, whereas fatty fish may contain polychlorinated biphenyls (PCBs) in addition to omega-3 fatty acids and vitamin D. Additionally, the absorption rate of protein rich food items may vary and is likely to affect postprandial energy metabolism (Boirie et al., 1997; Soucy and Leblanc, 1998; Stanstrup et al., 2014; Aadland et al., 2015, 2016; Vincent et al., 2017). To further complicate the picture, the effects of protein rich food on energy metabolism is strongly influenced by the background diet, i.e., the complemental part of the meal that also varies in micro- and macronutrients depending on food habits and culture. Finally, the gut microbiota may directly determine to what extent dietary proteins are converted into other metabolically active compounds such as short-chain fatty acids, branched chain fatty acids, or different nitrogen containing compounds (Lin et al., 2017).

Here, we summarize evidence of how different protein sources affect energy efficiency, obesity development and the gut microbiota in humans and mice. The overall phylum level composition in mouse gut is quite similar to that of the human gut microbiome, with Firmicutes, Bacteroidetes, and Proteobacteria comprising more than 70% of the gut microbiota (Xiao et al., 2015). Although only 4.0% of the mouse gut microbial genes are shared with those of the human gut microbiome, the mouse and human gut microbiome are functionally similar sharing 95.2% of their KEGG orthologous groups (Xiao et al., 2015). Of note, introduction of fecal microbes from human adult female twin pairs discordant for obesity into germ-free mice fed low-fat mouse chow induced an obese phenotype in chow fed mice receiving fecal bacteria from the obese twin (Ridaura et al., 2013). Vice versa, *Christensenella minuta*, a cultured member of the *Christensenellaceae* taxa known to be enriched in humans with low body mass index, led to reduced adiposity when transplanted to germ free mice together with an obese-associated microbiome (Goodrich et al., 2014). The reduced abundance of *Bacteroides thetaiotamicron* in obese humans is correlated with an increase in plasma glutamate, and interestingly, gavaging normal SPF C57BL/6 with *B. thetaiotaomicron* reduced diet-induced obesity in mice and diminished plasma glutamate levels (Liu et al., 2017). Hence, adiposity related phenotypes can be transmissible from humans to mice. We here review how different protein sources affect energy efficiency, obesity development and the gut microbiota. Finally, we discuss the possible importance of different amino acids, fatty acid composition and persistent organic pollutants (POPs). Different protein sources will also contain different amount of other food components, including micronutrients, trace elements, and vitamins. Their impact on the gut microbiota was recently reviewed (Roca-Saavedra et al., 2017), but since their possible roles on obesity development remain to be established, this will not be further described here.

## Protein sources, gut microbiota, and obesity

High protein diets represent a popular dietary approach to induce weight loss in humans. Data from numerous convincing rodent trials demonstrate strong attenuation or total prevention of high fat diet-induced obesity when the diet contains a high protein:carbohydrate ratio (Lacroix et al., 2004; Marsset-Baglieri et al., 2004; Morens et al., 2005; Pichon et al., 2006; Madsen et al., 2008; Ma et al., 2011; Freudenberg et al., 2012, 2013; Hao et al., 2012; McAllan et al., 2014). Still, review of human trials has revealed that the long-term effects of high-protein diets on obesity are neither consistent nor conclusive (Lepe et al., 2011; Astrup et al., 2015). In mice, we have demonstrated that long term high fat, high protein feeding limits obesity development and prevents the reduction in survival observed in mice fed a high fat, high sucrose diet compared to low fat fed mice (Kiilerich et al., 2016). In humans aged 50–65, results from the study of Levine et al. suggest that a high protein intake may be associated with increased overall mortality. However, this was not observed if the proteins were plant derived. It was suggested that the intake of animal proteins was a main factor driving the increase in overall mortality, highlighting the importance of the protein source (Levine et al., 2014) and the type of dietary protein would most likely modulate the (anti)-obesogenic effect of high protein diets also in humans.

The gut microbiota may also convert components from different protein sources into compounds that may be linked to development of disease. Well-known examples are L-carnitine and phosphatidylcholine, present in red meats and egg, which can be metabolized to trimethylamine and trimethylamine oxide (TMAO). In humans, circulating TMAO levels are reported to be associated with increased risk for atherosclerosis development (Koeth et al., 2013; Tang et al., 2013) and recently also linked to obesity (Dumas et al., 2017). However, fish and seafood, known to protect against cardiovascular disease contain high amounts of TMAO (Cho et al., 2017; Landfald et al., 2017). It is speculated that free TMAO from seafood may act as an electron acceptor for facultative anaerobic bacteria able to respire with the aldehydes resulting from the TMA elimination reactions of choline and carnitine (Cho et al., 2017).

Both diet composition (Arumugam et al., 2011; Graf et al., 2015; Kiilerich et al., 2016; Xiao et al., 2017) and the state of obesity (Ley et al., 2005, 2006) are important factors determining the gut microbial diversity in both animals and humans. As mentioned above, epidemiological studies have linked food groups and dietary patterns to obesity. Still, the linkage between diet, obesity, and gut microbiota is not elucidated. Vegetarian diets, whole grain products, fruits and nuts, vegetables, and legumes, as well as food constituents such as fat, protein, phytochemicals and fibers impact the gut microbiota in humans (Graf et al., 2015). The latter may be of particular importance as non-digestible carbohydrates or fibers represent a primary energy source for many gut microbes. Hence, bacterial fermentation, total bacterial numbers and species composition are affected. Further, fibers reduce the energy density of the diet, and microbial fermentation results in production of short chain fatty acids (SCFAs) that apart from a multitude of effects on host physiology may influence directly on metabolism (Flint, 2012; Koh et al., 2016; Sawicki et al., 2017). Accumulating evidence suggests the gut microbiota as a link between the dietary impact and host metabolism. The diet-induced response of the gut microbiota has been divided into; (1) a rapid gut response upon large dietary changes, (2) long-term dietary habits as a dominant factor for gut microbiota composition and (3) different responses upon dietary change due to individualized gut microbiota (reviewed in Sonnenburg and Backhed, 2016).

It has been reported that a high-protein low carbohydrate diet when given in combination with caloric restriction in obese humans resulted in an increase in branched-chain fatty acids, a decrease in butyrate, and a decrease in the abundance of the *Roseburia/Eubacterium rectale* group of bacteria in the gut (Russell et al., 2011). Concomitantly, the levels of metabolites associated with protection from cancer decreased whereas the levels of several metabolites assumed to be hazardous increased. Unfortunately, the protein sources in these diets were not described. Using casein as the protein source, we have demonstrated that in mice fed high fat diets with high or low protein:sucrose ratio, the dietary fat content, and not the protein:sucrose ratio, was the major driver of the composition of the gut microbiota (Kiilerich et al., 2016). Distinct changes in the gut microbiome composition were also observed over time, suggesting that host age in mice (Kiilerich et al., 2016) as well as in humans (Claesson et al., 2012) is also an important factor in defining the gut microbiome.

**Figure 1:**
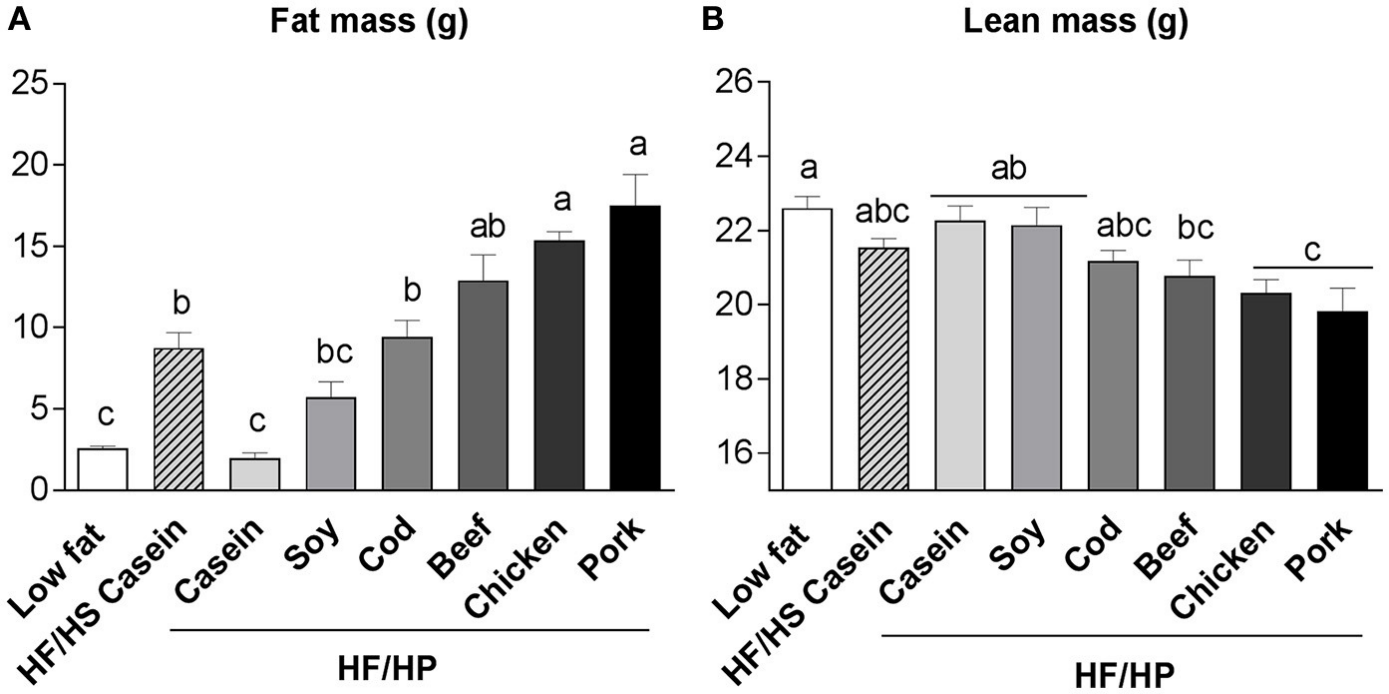
Fat mass **(A)** and lean mass **(B)** of male C57BL/6J mice fed high fat, high protein diets with different protein sources; casein, soy, cod, beef, chicken, or pork for 11 weeks at thermoneutral conditions. Mice fed a low fat and high fat, high sucrose diet based on casein were used as references. Diet composition and analyses are described in Liisberg et al. (2016b). Data represent mean ± SEM (*n* = 9). Different letters above the bars denote significant differences between the groups (*p* < 0.05), using 1-way ANOVA followed by Tukey's multiple comparison.

We have investigated the effect of a high fat, high protein diet using casein, soy, or filets of cod, beef, chicken (skinless) or pork as protein sources in C57BL/6J mice (Liisberg et al., 2016b). Of note, among the protein sources tested, casein was the only protein that actually prevented development of obesity and the mice fed casein had significantly lower fat mass than mice fed a high fat, high sucrose (HF/HS) reference diet (Figure 1; Liisberg et al., 2016b). Fat masses in mice fed a high proportion of soy, cod and beef were comparable to those fed HF/HS diet (Figure 1). Strikingly, compared with mice fed the HF/HS reference diet, mice fed diets with high content of proteins from chicken and pork had significantly more fat mass (Figure 1), also verified by dissection of different adipose tissue depots (Liisberg et al., 2016b). Mice fed proteins from pork exhibited a “whitening” of the classic brown adipose tissue, whereas the mice fed casein maintained a classic brown adipocyte morphology. The casein fed mice further exhibited an upregulated expression of genes related to futile cycling in brown adipose tissue. Of note, differences in obesity development could not be explained by differences in feed intake or digestibility of protein or fat (Liisberg et al., 2016b). Compared with mice fed a low fat reference diet and mice fed a high proportion of casein or soy, the pork and chicken fed mice also had reduced lean mass (Figure 1).

So far, very little is known on how different protein sources affect the gut microbiota. But recently, the composition of gut bacteria in the caecum of rats fed proteins from red meat (beef and pork), white meat (chicken and fish) as well as casein and soy was determined (Zhu et al., 2015). In this study, the meat proteins had been extracted from animal muscles and the animal feed contained regular amount (20%) of protein and was low (7%) in fat. Marked inter- and intra-group variation in the composition of the cecal microbiota was observed, with a more tight microbiota clustering of rats fed the non-meat proteins casein and soy, indicating that the composition of the gut microbiota diverged between rats fed feed based on meat proteins and non-meat proteins (Zhu et al., 2015). Young rats fed a diet with proteins from chicken (17.7%) for 14 days had an increased relative abundance in the genus *Lactobacillus*. However, the opposite pattern was demonstrated in middle-aged rats (Zhu et al., 2016).

Animal studies from our laboratories suggest that proteins from seafood are less obesogenic than proteins from terrestrial animals. C57BL/6J mice fed a Western diet containing a mixture of lean seafood (ling, rosefish, cod, wolf fish, and muscle from Canadian scallop) for 12 weeks accumulated less fat mass than mice fed a Western diet containing a mixture of skinless chicken breast, pork tenderloin, and beef sirloin (Holm et al., 2016). Comparison of the gut microbiomes of mice fed the two Western diets revealed significant differences in the relative abundance of operational taxonomic units belonging to the orders *Bacteroidales* and *Clostridiales*, with genes involved in metabolism of aromatic amino acids exhibiting higher relative abundance in the microbiomes of mice fed the seafood Western diet (Holm et al., 2016). Whether these differences in the abundance of microbial genes were of physiological relevance is still not clear. In a similar dietary setting, obesity development was attenuated in mice by exchanging lean pork meat with cod (Liisberg et al., 2016a). Further, intake of lean seafood such as white crab meat, scallop, and a mixture of cod and scallops has been demonstrated to attenuate diet-induced obesity and hepatic steatosis in mice (Tastesen et al., 2014a,b).

As discussed above, rodent studies clearly demonstrate that the protein source modulates the obesogenic effect of high protein diets and that casein is a rather unique protein source. However, the anti-obesogenic properties of casein may also extend to other milk-derived proteins, such as whey, and may be of relevance in humans. Epidemiological studies indicate that a high intake of low-fat dairy products is associated with protection against obesity (Fogelholm et al., 2012; Mozaffarian, 2016). Additionally, some human intervention studies using a dairy protein source with 80% casein and 20% whey have shown promising effects on weight loss (Zemel et al., 2004; Faghih et al., 2011). Still a meta-analysis of randomized controlled trials suggests that dairy intake only increases weight loss in combination with energy restriction (Chen et al., 2012).

To our knowledge, data on the potential of casein and/or whey to induce weight loss in obese animals is lacking. When present in the diet in regular amounts we have demonstrated that casein has an anti-obesogenic effect when compared with meat proteins from terrestrial animals and seafood (Ibrahim et al., 2011; Tastesen et al., 2014b). However, data from us and others suggest that whey is slightly more efficient than casein (Lillefosse et al., 2014; McAllan et al., 2015; Tranberg et al., 2015). Of note, concomitant with reduced weight gain, mice fed whey protein isolate had lower stomach weight and intestinal length (McAllan et al., 2015). In a study where mice were fed a high fat diet with either casein or a lactoperoxidase and lactoferrin-enriched whey protein isolate (WPI) at different doses, WPI specifically increased *Lactobacillaceae/Lactobacillus* and decreased *Clostridiaceae/Clostridium* in high fat diet-fed mice (McAllan et al., 2015). Further, Shi et al. have shown that replacing 5, 50, or 100% of the dietary casein-derived energy content with WPI caused a proportional suppression of body weight gain in high fat diet fed mice (Shi et al., 2011). It has been demonstrated that in comparison to casein, whey protein intake increased levels of *Lactobacilli* and *Bifidobacteria* in a rat model of colitis (Sprong et al., 2010). Still, whether the observed changes in the gut microbiota can be linked to differences in the anti-obesogenic effects of casein and whey remain to be investigated. It should be mentioned that some strains of *Bifidobacteria* are reported to have anti-obesogenic properties in rodents (An et al., 2011; Wang et al., 2015; Li et al., 2016). Increased proportions of *Lactobacillus* have been observed in high fat diet fed mice (Clarke et al., 2013). However, certain species of *Lactobacillus*, such as *Lactobacillus plantarum* (Isokpehi et al., 2017) were recently associated with weight-loss in humans. Further, supplementation with *Lactobacillus curvatus HY7601* and *L. plantarum KY1032* in diet-induced obese mice was associated with gut microbial changes and reduction in obesity (Park et al., 2013). The two probiotic strains *L. plantarum* KY1032 and *L. curvatus* HY7601 have also been demonstrated to reduce adipose tissue mass in diet-induced obese mice (Yoo et al., 2013).

In line with epidemiological studies indicating that a high intake of proteins from vegetarian sources and dairy is associated with protection against obesity (Fogelholm et al., 2012; Mozaffarian, 2016), rats receiving soy as the protein source were demonstrated to gain less body weight than rats receiving beef, pork, or turkey (Brandsch et al., 2006). A few human studies have investigated differences between omnivores and vegetarians but the results from these studies are not consistent (Graf et al., 2015). Human and animal studies investigating the effect of soy on the microbiota were recently reviewed, and in general, consumption of soy foods appeared to increase the levels of *Bifidobacteria* and *Lactobacilli* and alter the ratio between Firmicutes and Bacteroidetes (Huang et al., 2016). Although challenged, a decreased Bacteroidetes-to-Firmicutes ratio has been associated with obesity in both humans and animals (Ley et al., 2005; Turnbaugh et al., 2006). Further, as demonstrated with certain strains of *Lactobacillus* (see above), also some strains of *Bifidobacteria* have been associated with protection against diet-induced obesity in rodents (An et al., 2011; Wang et al., 2015; Li et al., 2016). Still, further studies are required to establish a causal relationship.

## Branched chain amino acids

Beyond the needs required for protein synthesis, amino acids participate in numerous pathways and certain amino acids may be directly involved in regulating metabolism. The impact on metabolism can be directly proportional to dietary intake. For instance, dietary intake of tryptophan or phenylalanine affects appetite regulation, intake of arginine alters nitric oxide production, and intake of branched chain amino acids (BCAAs) activates mammalian target of rapamycin complex 1. Casein and whey have a high content of the BCAAs; valine, leucine, and isoleucine. Of note, the chronic elevated levels of BCAAs in mice with disrupted mitochondrial branched chain aminotransferase were associated with increased energy expenditure (She et al., 2007). In rats fed a high fat diet, inclusion of BCAAs also attenuated obesity (Newgard et al., 2009). The relative high amounts of BCAAs in casein and whey, may hence contribute to the anti-obesogenic effect of dairy proteins. Studies by Freudenberg et al. have demonstrated that increasing leucine content in a high fat diet with regular protein content to a level matching a diet with high whey content attenuated obesity development (Freudenberg et al., 2012, 2013). Of note, BCAA-supplementation in mice delayed age-associated changes in the gut microbiota (Yang et al., 2016). In addition, BCAA supplemented mice had higher abundance of *Akkermansia* and *Bifidobacterium* in the gut. This may be of importance as *Akkermansia muciniphilia* has been associated with protection against diet-induced obesity (Everard et al., 2013; Shin et al., 2014), and the same has been reported for some strains of *Bifidobacteria* (An et al., 2011; Wang et al., 2015; Li et al., 2016). Still, the finding that equimolar supplementation with alanine decreased body fat mass gain in a short-term mouse experiment similarly as leucine (Freudenberg et al., 2013; Petzke et al., 2014), suggests that at least some of the observed effects are not specifically related to leucine, but due to increased amino nitrogen consumption. Thus, emphasizing that the effect of BCAA on metabolism is complex and far from fully understood.

## Taurine

Compared to terrestrial protein sources, seafood protein is characterized by high levels of taurine (Spitze et al., 2003). Supplementation of taurine to the diet or drinking water has been shown to prevent diet-induced weight gain, adiposity, and steatosis in rodents (Nakaya et al., 2000; Chang et al., 2011; Nardelli et al., 2011). In mice, it has been demonstrated that taurine supplementation reduced the abundance of Proteobacteria, especially *Helicobacter* and led to increased SCFA content in feces (Yu et al., 2016). SCFA, mainly acetate, propionate and butyrate are produced from non-digestible carbohydrates and may enter the systemic circulation and directly affect metabolism. Thus, SCFAs generally have been reported to counteract obesity in both rodents and humans (Canfora et al., 2015). However, a recent study revealed that increased acetate production led to hyperphagia and obesity in mice (Perry et al., 2016).

We have demonstrated that in mice fed an obesogenic diet with varying taurine concentrations, i.e., chicken, cod, crab, and scallop, for 7 weeks, the intake of taurine and glycine correlated negatively with body mass and total fat mass gain (Tastesen et al., 2014a). Further, rats fed diets with fish protein hydrolysate, rich in taurine and glycine exhibited reduced adiposity, possibly via increased bile acid concentration in plasma (Liaset et al., 2009, 2011). Primary bile acids are synthesized in the liver from cholesterol where they are conjugated to glycine or taurine in a species-dependent manner, and metabolized into secondary bile acids in the gut by the microbiota. Bile acids are ligands for the nuclear receptor farnesoid X receptor (FXR) and intestinal FXR is required for the development of obesity in response to a high-fat diet (Li et al., 2013). In addition, bile acids may also by binding and activating TGR5 increase energy expenditure (Watanabe et al., 2006; Broeders et al., 2015; Zietak and Kozak, 2016). In rodents, it has been proposed that the gut microbiota, in addition to regulating secondary bile acid metabolism, also inhibits bile acid synthesis in the liver by alleviating FXR inhibition in the ileum via reduced production of tauro-beta-muricholic acid (Sayin et al., 2013). Further, it is demonstrated that the gut microbiota promoted weight gain in an FXR-dependent manner, and the bile acid profiles and composition of fecal microbiota differed between *Fxr*^−/−^ and wild-type mice (Parseus et al., 2017). Hence, whether the amino acid composition in protein rich food is able to influence obesity development via effects on bile acid metabolism in the microbiota warrants further investigation.

## Fatty acid composition

In addition to the amino acid composition, different protein sources vary in other macronutrients. Regarding obesity development, the type and amount fat may be of particular importance. Meat from terrestrial sources, in particular red and processed meats, is rich in saturated fatty acids (SFAs), whereas meat from seafood, such as mackerel, halibut and salmon contains marine *n*-3 polyunsaturated fatty acids. The dietary fatty acid composition may influence obesity development directly, as well as via their reported influence on the gut microbiota. Animal studies have shown that intake of diets rich in SFAs resulted in increased adiposity and lower metabolic rate, relative to a polyunsaturated fatty acid (PUFA)-rich diet (Matsuo et al., 1995; Takeuchi et al., 1995). Data from an intervention study with abdominally obese humans also showed that intake of SFAs, as compared to *n*-6 PUFAs, promoted hepatic fat deposition (Bjermo et al., 2012). In an overeating study with young healthy adults, intake of SFAs induced hepatic and visceral fat gain as compared to intake of *n*-6 PUFAs (Rosqvist et al., 2014). Thus, consumption of SFAs is likely to induce fat gain relative to *n*-6 PUFAs. We recently demonstrated that the fatty acid composition in salmon filet may be of importance. Replacement of marine oils in salmon feed with vegetable oils, soybean oil in particular, profoundly increased the *n*-6:*n*-3 ratio in fish filets and in red blood cells (RBCs) collected from mice consuming the salmon (Alvheim et al., 2013; Midtbo et al., 2013). Of note, the increased *n*-6:*n*-3 ratio in the RBC of the mice was accompanied with increased obesity (Alvheim et al., 2013; Midtbo et al., 2013, 2015). Although the results from rodent studies are more convincing than results from human trials (Madsen and Kristiansen, 2012), *n*-3 fatty acids are reported to increase weight loss also in humans (Thorsdottir et al., 2007).

It is well-known that the gut microbiota of high-fat diet-induced obese mice differs from that of lean mice (Serino et al., 2012; Xiao et al., 2015; Kiilerich et al., 2016), and we recently demonstrated that high-fat feeding rather than obesity drives taxonomical and functional changes in the gut microbiota in mice (Xiao et al., 2017). The dietary fatty acid composition is also reported to change the gut bacteria profile. Compared with *n*-6 or *n*-3 PUFAs, feeding mice diets rich in SFAs, over a 14-week period decreased the proportion of Bacteroidetes species and hence, the SFA-rich diet resulted in significantly greater decreases in Bacteroidetes-to-Firmicutes ratio than did either of the PUFA-rich diets (Liu et al., 2012). Mice fed fish oil are also reported to have increased levels of *A. muciniphila* (Caesar et al., 2015), which has been shown to reduce fat mass gain, and white adipose tissue macrophage infiltration, and to improve gut barrier function and glucose metabolism when administered to mice with diet-induced obesity. Further, antibiotic-treated mice receiving gut microbiota from a lard-fed donor showed increased adiposity and inflammation, whereas enrichment of *Akkermansia* co-occurred with partial protection against adiposity in mice transplanted with microbiota from fish oil fed mice (Caesar et al., 2015).

The different fatty acids have different capacities to activate the Toll-like receptors. In the study mentioned above, saturated lipids from lard were suggested to induce metabolic inflammation through Toll-like receptor signaling mediated by the gut microbiota (Caesar et al., 2015). By contrast, in another study on middle-aged rats, fish oil feeding was demonstrated to increase the relative abundances of the phylum Proteobacteria and the genus *Desulfovibrio*, concurrent with induced inflammation compared to rats receiving diets with soy bean oil or lard (Li et al., 2017). The innate pathogen receptors, a part of the first line of defense against infectious agents, including Toll-like receptors, nucleotide oligomerization domain containing proteins, and inflammasomes, have been pointed out as a link between the gut microbiota and host metabolism (Jin and Flavell, 2013). Inflammasome-deficiency-dependent modulation of the gut microbiota may be associated with abnormalities related to the metabolic syndrome and obesity in mouse models (Henao-Mejia et al., 2012). Further, changes in gut permeability may be affected through the interaction between diet, host and gut microbiota, augmenting access for proinflammatory molecules and activating inflammation, thereby affecting obesity development (reviewed in Tremaroli and Backhed, 2012).

It has been demonstrated that inclusion of lean fish in low energy diets was as efficient as inclusion of fatty fish or fish oil supplement in accelerating weight loss in humans (Thorsdottir et al., 2007). The total content of *n*-3 PUFAs is far lower in lean than in fatty seafood, but in lean seafood the majority of the fatty acids are present in the phospholipid (PL) fraction (Lie and Lambertsen, 1991). The bioavailability of eicosapentaenoic acid (EPA) and docosahexaenoic acid (DHA) is reported to be higher when they are PL-bound than triacylglycerol (TAG)-bound (Murru et al., 2013). Of note, the anti-obesogenic effects of PL-bound *n*-3 PUFAs are superior to TAG-bound *n*-3 PUFAs in mice (Rossmeisl et al., 2012). The high biological activity of PL-bound PUFAs is suggested to include effects mediated via the endocannabinoid signaling system (Batetta et al., 2009; Rossmeisl et al., 2012).

The endocannabinoid signaling system may link the gut microbiota to adipogenesis, as CB_I_ receptors are reported to control gut permeability through interactions with the gut microbiota (Muccioli et al., 2010). Further, changes in the composition of the gut microbiota during obesity induce gut-barrier dysfunction, which may lead to leakage from the gut of components from Gram-negative bacteria and metabolic endotoxemia triggering the onset of metabolic disorders associated with obesity (Cani et al., 2016). Conversely, administration of the intestinal bacterium *A. muciniphila* to high-fat diet fed mice led to an increase in intestinal levels of the endocannabinoids, 2-arachidonoylglycerol, 2-oleoylglycerol and 2-palmitoylglycerol, along with improved gut-barrier function and decreased metabolic endotoxemia (Everard et al., 2013). The molecular mechanisms that link gut microorganisms and the synthesis of endocannabinoids or other bioactive lipids are still unknown, and it is not known to what extent such molecules play significant roles in obesity development or metabolic dysfunctions associated with obesity.

## Persistent organic pollutants

Importantly, food is also the primary route of exposure to pollutants from numerous chemical classes. Actually, food contributes more than 90 % to the total current exposure of POPs, especially food of animal origin such as fish, dairy products, or meat (Li et al., 2006; Malisch and Kotz, 2014). During the last decade increasing attention has been paid to the possible relationship between POP exposure and the current obesity epidemic. Studies have reported an association between obesity and plasma levels of certain polychlorinated biphenyls (PCBs) and pesticides (Dhooge et al., 2010; Ronn et al., 2011; Lee et al., 2012; Roos et al., 2013). Thus, concern about the possible connection between exposure to environmental contaminants and the epidemics of obesity has been raised (Lee et al., 2014). However, a causal relationship between POP exposure and obesity development has not yet been demonstrated, and inverse relationships between obesity and plasma levels of POPs, in particular highly chlorinated PCBs, are also reported (Nawrot et al., 2002; Dirinck et al., 2011). A direct causal link between POP exposure and obesity in humans is difficult to establish, but repeated injections of PCB-153 (Wahlang et al., 2013) and PCB-77 (Arsenescu et al., 2008) are reported to exacerbate obesity in mice. We have previously observed that POPs of marine origin accumulate in adipose tissue concomitant with obesity development in mice fed farmed Atlantic salmon (Ibrahim et al., 2011). However, mice fed high levels of POPs from whale meat were leaner than control casein fed mice, despite a high accumulation of POPs in adipose tissue (Ibrahim et al., 2012). In a recent study we reduced the levels of PCBs and DDTs by 50% in salmon filets by partial replacement of fish oil with vegetable oils in aquatic feed leading to aggravated insulin resistance and hepatic lipid accumulation, despite a reduction in the levels of PCBs and DDTs (Midtbo et al., 2013).

The relationship between the gut bacteria and environmental pollutants is bidirectional (Parks et al., 2013). First, POP exposure alters the composition of the gut microbiota in mice. It has been demonstrated that gavage with a high dose of a mixture of PCB congeners found in meat and fatty fish for 2 days diminished the overall abundance of bacteria (Choi et al., 2013). Of note, exercise prevented these changes (Choi et al., 2013). Further, it has been reported that exposure to 2,3,7,8 tetrachlorodibenzofuran, TCDF, alters the gut microbiome by shifting the ratio of Firmicutes to Bacteroidetes (Zhang et al., 2015), which as mentioned earlier has been associated with obesity (Ley et al., 2005; Turnbaugh et al., 2006). Secondly, the gut microbiota can affect body burden of POPs by a number of mechanisms. The bacteria may metabolize the POPs and alter absorption and excretion as well as influence the host detoxification capacity and the enterohepatic circulation of environmental chemicals (Claus et al., 2016; Spanogiannopoulos et al., 2016). Last, we have provided evidence that the dietary composition of macronutrients profoundly modulates accumulation of four environmental relevant POPs in adipose tissue and liver in C57BL/6 mice (Myrmel et al., 2016). Thus, understanding the potential roles of the gut microbiota in the metabolism and toxicity of environmental pollutants could provide valuable knowledge for targeted approaches in reducing the detrimental health impact of environmental pollution.

## Conclusion

**Figure 2:**
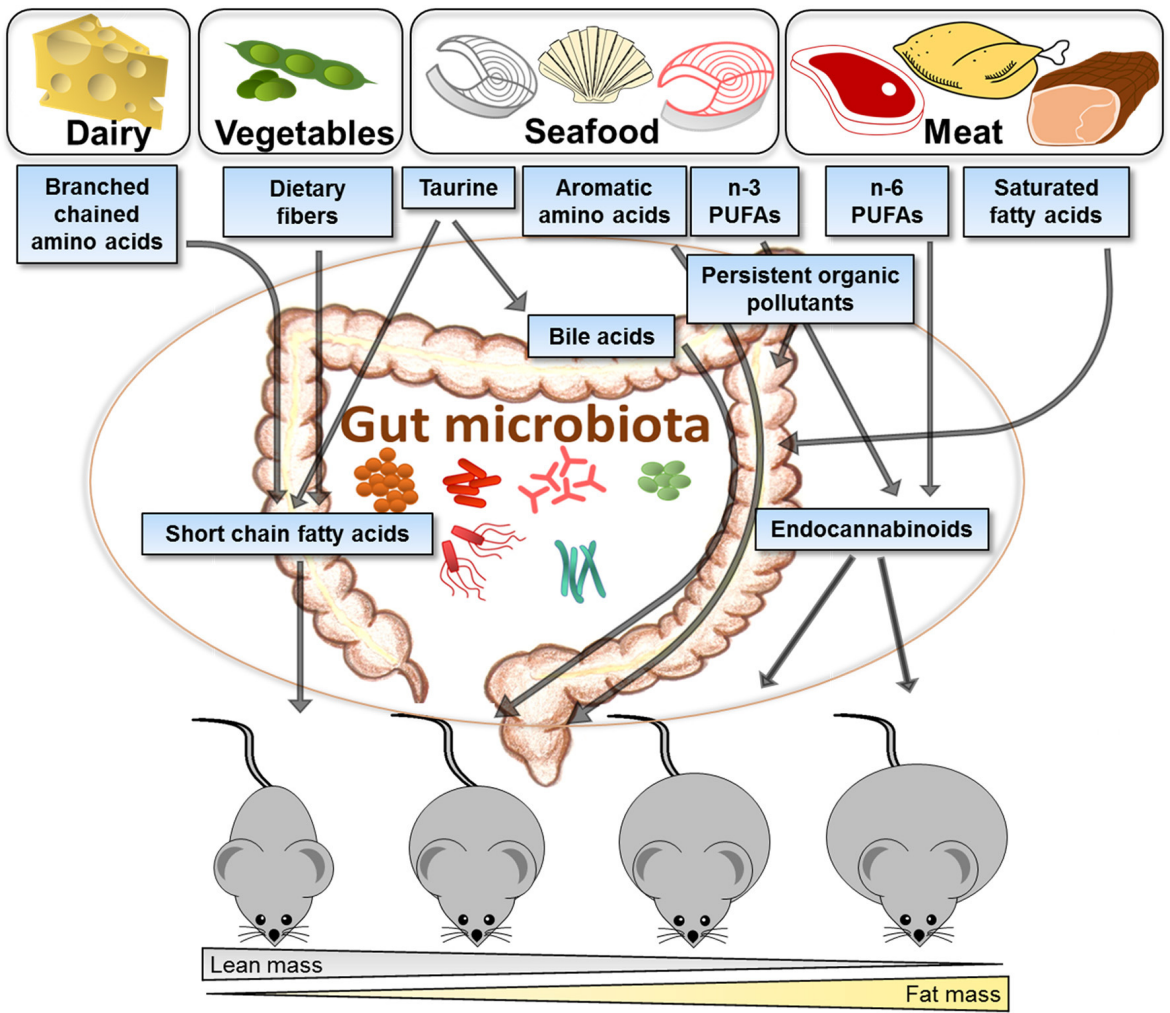
Illustration of how different protein sources vary in their efficiency to attenuate obesity development and suggested links with the gut microbiota. Proteins derived from different food sources contain varying amounts of amino acids, fatty acids, and pollutants, which may interact with the gut microbiota and change the host metabolism, and further impact on obesity development. Casein and other dairy products have a high content of branched chain amino acids and are efficient protein sources for attenuating obesity development in rodents. Proteins from vegetarian sources contribute to high fiber content in the diet and have been demonstrated to protect against obesity. Compared to animal protein sources, seafoods contain high amounts of taurine, aromatic amino acids, *n*-3 polyunsaturated fatty acids (PUFAs) and persistent organic pollutants, which further may impact on the gut microbiota, production of bile acids or endocannabinoids. Different sources of meat contribute with saturated fatty acids, n-6 PUFAs and persistent organic pollutants. Generally, intake of proteins from meat has been demonstrated to be more obesogenic than intake of proteins from seafood or vegetables. It remains to be established to what extent such differences between proteins reflect direct metabolic effects in the host or to what extent the microbiota plays a causal role.

The efficiency of different proteins to attenuate obesity in both human and animal trials varies depending on the protein source as illustrated in Figure 2. Different food groups and dietary patterns are linked to obesity and disease development, and to distinct gut microbiota composition. However, the association between diet, obesity and gut microbiota is not established. Here we have reviewed data from several different studies in order to illustrate how different protein sources may affect the gut microbiota, linking protein-dependent changes in the gut microbiota with metabolism. The varying amounts of amino acids, fatty acid composition, and POPs derived from different protein sources, such as dairy, vegetarian sources, seafood and meat, are discussed in light of their impact on gut microbiota and obesity. Casein and other dairy proteins stand out as the most efficient protein sources in preventing obesity, having a high content of BCAAs with possible impact on gut microbiota and obesity. The distinct impacts of lean seafood and meat are suggested to involve differences in the content of amino acids, such as aromatic amino acids, glycine and taurine, possibly affecting the bile acids with further impact on energy expenditure and the gut microbiota. The *n*-3 PUFAs may affect the metabolism through the endocannabinoid system and the gut microbiota composition, but the molecular mechanisms linking the gut microorganisms to the synthesis of endocannabinoids or other bioactive lipids are still largely unexplored. As dietary sources will inevitably lead to exposure of pollutants, their impact on metabolism may also contribute to the varying impact of different protein sources. Knowledge of the mutually reciprocal interactions between the gut microbiota and environmental pollutants present in different food sources and the combined effects on whole body metabolism could provide new modalities involving targeted dietary approaches reducing the detrimental health impact of environmental pollution.

## Author contributions

LM: Researched data and wrote the article; KK, LSM, EF, and BL: Contributed to discussion of the content, illustration, revised, and/or edited the manuscript before submission.

### Conflict of interest statement

The authors declare that the research was conducted in the absence of any commercial or financial relationships that could be construed as a potential conflict of interest.
